# Supplementary material for: ClpAP proteolysis does not require rotation of the ClpA unfoldase relative to ClpP
Source: eLife. 2020 Dec 1;9:e61451. doi: 10.7554/eLife.61451 (PMC7707817; doi:10.7554/eLife.61451)
Supplement: Figure 2—source data 1. — Values are relative fluorescent units (RFUs) calculated from the area under the curve of each species band on a Coomassie-stained SDS-PAGE gel. The volume of samples (3 or 5 µL) loaded in each lane is indicated and used to generate two standard curves for each sample load volume. No RFUs were recorded for the E613CClpA‡ samples containing only uncrosslinked ClpA that were used for the standard curves (represented by N/A). Concentrations of ClpA were calculated from the standard curve with matching sample load volumes. [file elife-61451-fig2-data1.docx]

**Figure 2—source data 1 – Quantification of A–P crosslinking efficiency**

Values are relative fluorescent units (RFUs) calculated from the area under the curve of each species band on a Coomassie-stained SDS-PAGE gel. The volume of samples (3 or 5 µL) loaded in each lane is indicated and used to generate two standard curves for each sample load volume. No RFUs were recorded for the ^E613C^ClpA^‡^ samples containing only uncrosslinked ClpA that were used for the standard curves (represented by N/A). Concentrations of ClpA were calculated from the standard curve with matching sample load volumes.

| **Sample** | RFUs A**–**P band | RFUs ^E613C^ClpA^‡^ band |
| --- | --- | --- |
| 3 µL load, 11.4 µM ^E613C^ClpA^‡^ | N/A | 381542.6 |
| 3 µL load, 5.57 µM ^E613C^ClpA^‡^ | N/A | 188451.7 |
| 3 µL load, 2.84 µM ^E613C^ClpA^‡^ | N/A | 97512.08 |
| 3 µL load, 1.22 µM ^E613C^ClpA^‡^ | N/A | 50215.5 |
| 3 µL load, 0.61 µM ^E613C^ClpA^‡^ | N/A | 30570.5 |
| 3 µL load, undiluted A**–**P pool | 129781.5 | 23502.5 |
| 3 µL load, 2-fold diluted A**–**P pool | 65359 | 11884.78 |
| 5 µL load, 11.4 µM ^E613C^ClpA^‡^ | N/A | 699406 |
| 5 µL load, 5.57 µM ^E613C^ClpA^‡^ | N/A | 322593 |
| 5 µL load, 2.84 µM ^E613C^ClpA^‡^ | N/A | 177321 |
| 5 µL load, 1.22 µM ^E613C^ClpA^‡^ | N/A | 86503.78 |
| 5 µL load, 0.61 µM ^E613C^ClpA^‡^ | N/A | 43850 |
| 5 µL load, undiluted A**–**P pool | 190351 | 28275.5 |
| 5 µL load, 2-fold diluted A**–**P pool | 97501 | 19058 |
