# Supplementary material for: ClpAP proteolysis does not require rotation of the ClpA unfoldase relative to ClpP
Source: eLife. 2020 Dec 1;9:e61451. doi: 10.7554/eLife.61451 (PMC7707817; doi:10.7554/eLife.61451)
Supplement: Figure 3—source data 1. — Values are means of fraction remaining of λ cIN-ssrA from three technical replicates ± 1 SD. Values were not recorded (NR) for A–P at 8 min. [file elife-61451-fig3-data1.docx]

**Figure 3—source data 1 – Quantification of λ cI^N^-ssrA degradation kinetics**

Values are means of fraction remaining of λ cI^N^-ssrA from three technical replicates ± 1 SD. Values were not recorded (NR) for A–P at 8 min.

| Time (min) | A•P | A–P |
| --- | --- | --- |
| 0 | 1.0 ± 0 | 1.0 ± 0 |
| 5 | 0.28 ± 0.08 | 0.74 ± 0.05 |
| 8 | 0.08 ± 0.02 | NR |
| 10 | 0.01 ± 0.00 | 0.56 ± 0.09 |
| 15 | 0.00 ± 0 | 0.33 ± 0.10 |
| 20 | 0.00 ± 0 | 0.20 ± 0.07 |
| 30 | 0.00 ± 0 | 0.06 ± 0.05 |
