# Supplementary material for: ClpAP proteolysis does not require rotation of the ClpA unfoldase relative to ClpP
Source: eLife. 2020 Dec 1;9:e61451. doi: 10.7554/eLife.61451 (PMC7707817; doi:10.7554/eLife.61451)
Supplement: Figure 3—source data 2. — In columns 2–3, degradation rates are mean degradation rates from 3 to 4 technical replicates ±1 SD. In column 4, the fractional rates were calculated from dividing the mean A–P degradation rate by the mean A•P degradation rate, and the error is a propagated error calculated using the following formula. [file elife-61451-fig3-data2.docx]

**Figure 3—source data 2– Degradation of substrates of varying thermodynamic stability**

In columns 2-3, degradation rates are mean degradation rates from 3-4 technical replicates ± 1 SD. In column 4, the fractional rates were calculated from dividing the mean A–P degradation rate by the mean A•P degradation rate, and the error is a propagated error calculated using the following formula:

$$propagated error for fractional rate=\frac{rate A-P}{rate A\bullet P}*sqrt\left( \left( \frac{Stdev A\bullet P}{rate A\bullet P} \right)^{2}+\left( \frac{Stdev A-P}{\mathrm{rate}A-P} \right)^{2} \right)$$

| **Protein substrate** | **A•P degradation rate (min^-1^ ClpA_6_^-1^)** | **A–P degradation rate**  **(min^-1^ ClpA_6_^-1^)** | **Fractional rate**  **(A­–P/ A•P)** | ***n*** |
| --- | --- | --- | --- | --- |
| FITC-casein | 7.34 ± 0.23 | 7.11 ± 0.44 | 0.97 ± 0.07 | 3 |
| ^5-IAF^V13P titin^I27^-ssrA | 5.56 ± 0.15 | 2.57 ± 0.07 | 0.46 ± 0.02 | 4 |
| ^cp7^GFP-ssrA | 2.72 ± 0.12 | 0.86 ± 0.11 | 0.32 ± 0.04 | 4 |
| λ cl^N^-ssrA | 1.93 ± 0.28 | 0.60 ± 0.05 | 0.31 ± 0.05 | 3 |
