# Supplementary material for: ClpAP proteolysis does not require rotation of the ClpA unfoldase relative to ClpP
Source: eLife. 2020 Dec 1;9:e61451. doi: 10.7554/eLife.61451 (PMC7707817; doi:10.7554/eLife.61451)
Supplement: Figure 3—source data 3. — Values are mean FITC-casein degradation rates from three technical replicates ± 1 SD. [file elife-61451-fig3-data3.docx]

**Figure 3—source data 3– Degradation of FITC-casein (18 µM) by the purified A–P pool in the presence of ATP or ATPγS**

Values are mean FITC-casein degradation rates from three technical replicates ± 1 SD.

| **Nucleotide** | **A–P rate**  **(substrate min^-1^ ClpA_6_^-1^)** |
| --- | --- |
| 4 mM ATP | 2.03 ± 0.30 |
| 4 mM ATPγS | 0.27 ± 0.06 |
