# Supplementary material for: ClpAP proteolysis does not require rotation of the ClpA unfoldase relative to ClpP
Source: eLife. 2020 Dec 1;9:e61451. doi: 10.7554/eLife.61451 (PMC7707817; doi:10.7554/eLife.61451)
Supplement: Figure 3—source data 4. — Values are mean degradation rates of cp7GFP-ssrA from three technical replicates ± 1 SD. Values were not recorded (NR) for A•P at 7.5 µM. [file elife-61451-fig3-data4.docx]

**Figure 3—source data 4 – Michaelis-Menten analysis of ^cp7^GFP-ssrA degradation kinetics**

Values are mean degradation rates of ^cp7^GFP-ssrA from three technical replicates ± 1 SD. Values were not recorded (NR) for A•P at 7.5 µM.

| **^cp7^GFP-ssrA Concentration (µM)** | **A–P**  **(min^-1^ ClpA6^-1^)** | **A•P**  **(min^-1^ ClpA6^-1^)** |
| --- | --- | --- |
| 0.31 | 0.03 ± 0.01 | 0.16 ± 0 |
| 0.63 | 0.06 ± 0.02 | 0.33 ± 0.02 |
| 1.25 | 0.13 ± 0.01 | 0.57 ± 0.02 |
| 2.5 | 0.23 ± 0.03 | 0.98 ± 0.05 |
| 5 | 0.36 ± 0.05 | 1.96 ± 0.07 |
| 7.5 | 0.44 ± 0.01 | NR |
| 10 | 0.69 ± 0.07 | 2.44 ± 0.22 |
| 20 | 0.86 ± 0.11 | 2.72 ± 0.12 |
| 40 | 1.22 ± 0.07 | 2.43 ± 0 |
| 80 | 1.12 ± 0.12 | 2.82 ± 0.14 |
