# Supplementary material for: ClpAP proteolysis does not require rotation of the ClpA unfoldase relative to ClpP
Source: eLife. 2020 Dec 1;9:e61451. doi: 10.7554/eLife.61451 (PMC7707817; doi:10.7554/eLife.61451)
Supplement: Figure 3—source data 5. — Values are mean ATP hydrolysis rates (5 mM ATP) or mean degradation rates (20 µM cp7GFP-ssrA) from three technical replicates ± 1 SD. [file elife-61451-fig3-data5.docx]

**Figure 3—figure supplement 1A-B—source data 5—ATPase and ^cp7^GFP-ssrA degradation rates by ClpAP controls**

Values are mean ATP hydrolysis rates (5 mM ATP) or mean degradation rates (20 µM ^cp7^GFP-ssrA) from three technical replicates ± 1 SD.

| **ClpAP variant** | **ATPase rate (min^-1^ ClpA_6_^-1^)** | **^cp7^GFP-ssrA degradation rate (min^-1^ ClpA_6_^-1^)** |
| --- | --- | --- |
| ClpA^WT^ ClpP^WT^ | 1007 ± 83 | 2.74 ± 0.11 |
| ClpA^CF^ ClpP^WT^ | 840 ± 82 | 2.52 ± 0.09 |
| A•P (^E613C^ClpA^‡^ ClpP^WT^) | 1035 ± 78 | 2.72 ± 0.12 |
| ClpA^WT^ ClpP^+C^ | 671 ± 60. | 2.23 ± 0.08 |
| ClpA^CF^ ClpP^+C^ | 787 ± 59 | 2.50 ± 0.13 |
| ^E613C^ClpA^‡^ ClpP^+C^ (no crosslinking) | 895 ± 33 | 2.75 ± 0.19 |
| A–P (^E613C^ClpA^‡^ crosslinked to P^+C^) | 412 ± 40. | 0.86 ± 0.11 |
